# *FBLN1* regulates ferroptosis in acute respiratory distress syndrome by reducing free ferrous iron by inhibiting the TGF-β/Smad pathway

**DOI:** 10.1371/journal.pone.0314750

**Published:** 2024-12-13

**Authors:** Yaping Yuan, Youbo Wang, Yufeng Yan, Edward Kim, Jin Bai, Yang Zhao, Qinyun Ma, Wenchao Gu, Haihan Song

**Affiliations:** 1 Department of Pulmonary and Critical Care Medicine, Shanghai Pudong New Area People’s Hospital, Shanghai, China; 2 Department of Thoracic Surgery, Huashan Hospital, Fudan University, Shanghai, China; 3 Department of Neurosurgery, Jinshan Hospital, Fudan University, Zhujing Town, Jinshan District, Shanghai, China; 4 Department of Immunology, DICAT National Biomedical Computation Centre, Vancouver, BC, Canada; 5 CRT Medical Union, Time International, Beijing, China; 6 Central Lab, Shanghai Key Laboratory of Pathogenic Fungi Medical Testing, Shanghai Pudong New Area People’s Hospital, Shanghai, China; The Second Affiliated Hospital of Guangzhou Medical University, CHINA

## Abstract

**Background:**

Acute respiratory distress syndrome (ARDS) / acute lung injury (ALI) is a serious medical disease characterized by pulmonary dysfunction and inflammation. This study aims to determine the main molecular modules linked to ARDS and investigate the role of Fibulin-1 (FBLN1) in regulating ferroptosis in ARDS.

**Methods:**

Weighted Gene Co-expression Network Analysis (WGCNA) was employed on the GSE263867 dataset to find key modules associated with ALI. Differentially expressed genes (DEGs) and protein-protein interaction (PPI) networks were analyzed. MLE-12 cells were treated with lipopolysaccharide (LPS) to induce ferroptosis. *In vitro* studies were conducted to investigate the effects of FBLN1 and Transforming Growth Factor Beta 1 (TGF-β) overexpression on cell viability, oxidative stress markers, and ferroptosis-related proteins.

**Results:**

WGCNA identified the turquoise module as significantly negatively correlated with ARDS. Five key overlapping genes (*GRIA1*, *OGN*, *COL14A1*, *FBLN1*, and *COL6A3*) were significantly downregulated in ARDS samples. LPS treatment induced ferroptosis in MLE-12 cells, indicated by increased malondialdehyde (MDA), lipid reactive oxygen species (ROS), and ferrous iron (Fe^2^⁺) levels, and decreased cell viability and glutathione (GSH) levels. FBLN1 overexpression partially reversed these effects. Additionally, FBLN1 inhibited the TGF-β/Smad signaling pathway, as shown by decreased TGF-β and p-Smad protein levels. TGF-β overexpression exacerbated LPS-induced oxidative stress and ferroptosis, reducing cell viability and GSH levels. FBLN1 overexpression counteracted this effect, suggesting antagonistic roles for FBLN1 and TGF-β in regulating ferroptosis.

**Conclusion:**

This study highlights *FBLN1* as a critical regulator of ferroptosis in ARDS. Targeting the TGF-β/Smad pathway to modulate *FBLN1* expression offers a potential therapeutic strategy to alleviate oxidative stress and mitigate pulmonary injury in inflammatory lung diseases.

## Introduction

Acute respiratory distress syndrome (ARDS) / acute lung injury (ALI) is a severe pulmonary disease marked by increased vascular permeability, diffuse alveolar damage, and acute respiratory distress [1]. A variety of etiological factors, including sepsis, pneumonia, trauma, and aspiration can trigger this condition [2]. The incidence of ARDS remains high, with morbidity and mortality rates often exceeding 40% [3]. Despite advances in understanding the pathophysiology of ARDS, current treatment options are primarily supportive, involving mechanical ventilation and conservative fluid management [4]. These interventions, however, have limited efficacy in improving long-term outcomes [5]. Given the substantial incidence and fatality linked to ARDS, there is a pressing requirement for the creation of new biomarkers for diagnosis, therapeutic strategies, and prognostic indicators to enhance patient management and improve clinical outcomes.

Fibulin-1 (FBLN1) is an extracellular matrix (ECM) protein involved in cell adhesion, migration, and tissue repair, playing a vital function in maintaining the structural integrity and function of different tissues, including the respiratory system [6,7]. In respiratory diseases, *FBLN1* is implicated in fibrosis and inflammation, key components of conditions like asthma and chronic obstructive pulmonary disease (COPD) [8]. Recent studies have highlighted the importance of *FBLN1* in regulating ferroptosis, a kind of iron-dependent lipid peroxidation-driven programmed cell death, which contributes to acute ALI by promoting oxidative stress and cellular damage [9,10]. Liu G et al. discovered that *FBLN1* is involved in the pathogenesis of both COPD and asthma. In COPD, increased levels of *FBLN1* are observed in patients and experimental models, with inhibiting *FBLN1c* reducing cigarette smoke-induced airway fibrosis, emphysema, and pulmonary inflammation [11]. Similarly, another study by Liu G et al. found that in asthma, *FBLN1c* stabilizes ECM proteins, and its elevated levels are associated with increased airway remodeling and inflammation [12]. Inhibiting *FBLN1c* in mouse models reduces airway collagen deposition and hyperresponsiveness. These outcomes imply that *FBLN1* may be a beneficial target for therapy for COPD and chronic asthma, providing a promising strategy for managing lung diseases.

Given the emerging role of *FBLN1* in regulating ferroptosis and its impact on respiratory diseases, this research intended to explore the potential guarding impacts of *FBLN1* overexpression against lipopolysaccharide (LPS)-induced ARDS. Specifically, we sought to elucidate the molecular mechanisms by which FBLN1 regulates ferroptosis and oxidative stress in lung epithelial cells. Using weighted gene co-expression network analysis (WGCNA), the overlapping genes and important modules linked to ARDS were found. We then evaluated the effects of FBLN1 and TGF-β overexpression on ferroptosis-related markers, oxidative stress, and cell viability in MLE-12 cells. Our conclusions offer perspective into the therapeutic potential of targeting *FBLN1* and the TGF-β/Smad pathway to mitigate ferroptosis and improve ARDS outcomes.

## Materials and methods

### Downloading and processing of the GSE263867 dataset

The GSE263867 dataset was downloaded and processed from the Gene Expression Omnibus (GEO, https://www.ncbi.nlm.nih.gov/gds/). Samples from ALI (n = 10) and comparable controls (n = 5) are included in this dataset. The data were preprocessed using R programming. For multiple probe sets corresponding to the same gene, the mean expression values were calculated. Probe IDs were translated into gene symbols. Differential expression analysis was conducted utilizing the Limma package in R. Fold change (FC) threshold > 2 was called up-regulated differentially expressed genes (DEGs), FC < 0.5 was called down-regulated DEGs, and p-value criterion with an adjustment was < 0.01.

### Weighted gene co-expression network analysis (WGCNA)

The network of co-expression for all genes in the GSE263867 dataset was constructed using the Bioinfo Intelligent Cloud website (https://www.bic.ac.cn/BIC/#/). Accurately adjusting the soft threshold power to β = 30 ensured scale-free topology. This threshold ensures that the network adheres to the properties of scale-free topology, where a few genes (hubs) exhibit high connectivity while most genes have low connectivity, which is a key feature of biological networks. Once the power was determined, an adjacency matrix was created by calculating the pairwise Pearson correlation coefficients between gene expression levels, then raising these correlations to the power of β to assign weights to the connections. This step highlights the strength of relationships between gene pairs. To refine the network, the adjacency matrix was transformed into a topological overlap matrix (TOM), which captures not only direct correlations but also the shared neighbors of genes. The TOM is a more robust measure of interconnectedness, as it accounts for both the direct and indirect relationships between genes, making it a reliable metric for determining gene co-expression similarity. A dendrogram was generated by applying hierarchical clustering to the TOM. Different colored branches in this structure correspond to different gene modules. Weighted correlation coefficients were employed to consolidate genes with analogous expression trajectories into appropriate modules. Eventually, we sought to ascertain the correlation between clinical traits and gene modules with the objective of identifying the key module.

### Identification of key overlapping genes and their expression analysis by bioinformatics

The Bioinformatics & Evolutionary Genomics platform was employed in the intersection analysis of DEGs (including down-regulated DEGs and up-regulated DEGs) in the GSE263867 dataset and genes in the turquoise module to identify overlapping genes. To elucidate potential protein-protein interactions (PPIs) among the overlapping genes, the Search Tool for the Retrieval of Interacting Genes (STRING, https://string-db.org/) database was applied for a PPI network analysis. The generated PPI network, including metrics such as degree, Maximal Clique Centrality (MCC), and Maximal Neighborhood Component (MNC), was visualized utilizing Cytoscape (Version 3.7.1). This visualization enabled a thorough examination of protein interactions. Further cross-analysis of genes from the three network modules was performed using the bioinformatics platform to obtain key overlapping genes. Data processing and box plot visualization were conducted using the Sangerbox website (Version 3.0, http://vip.sangerbox.com/home.html) to examine the expression of key overlapping genes in ALI samples and normal controls within the GSE263867 dataset. The statistical significance of the results was assessed with a threshold set at *p* < 0.05.

### Cell lines and culture

The MLE-12 cell line is a commonly used mouse alveolar epithelial cell line that is often used to model and study lung diseases, including ALI. The Shanghai Institute of Cell Biology, Chinese Academy of Sciences (Shanghai, China) provided the MLE-12 cells, which were then cultured in Dulbecco’s Modified Eagle Medium (DMEM) with 10% fetal bovine serum (FBS) and 1% penicillin-streptomycin as supplements. A humidified environment containing 5% CO_2_ was established to sustain cell cultures at 37°C.

### Cell treatment

To investigate the induction of ferroptosis, LPS was applied after the cells were seeded in 6-well plates at concentrations of 0.5, 1, 2, and 5 μg/mL for 6, 12, 24, and 48 hours. Ferrostatin-1 (Fer-1, 1 μM), an inhibitor of ferroptosis, and Erastin (Era, 3 μM), a ferroptosis inducer, were also used in separate treatment groups to assess their effects on ferroptosis in MLE-12 cells. The group with only DMSO added is the negative control group, Control.

### Transfection assay

For transient transfection, MLE-12 cells were seeded at a density of 2 × 10^5^ cells per well in 24-well plates. According to the manufacturer’s protocol, cells were transfected with plasmids encoding FBLN1 and TGF-β by Lipofectamine 3000 (Invitrogen, China). The transfection aimed to overexpress FBLN1 and TGF-β proteins to study their roles in ferroptosis and ALI. Specific small interfering RNAs (siRNAs) targeting *FBLN1* (si- *FBLN1* sequences: 5′-GCUGGAGAUGAACUACGTGTT-3′) were used to knock down *FBLN1* expression in the MLE-12 cells. A non-targeting siRNA (si-NC sequences: 5′-UUCUCCGAACGUGUCACGUTT-3′) served as a negative control. Transfection was carried out utilizing Lipofectamine 3000 (Invitrogen, China) according to the manufacturer’s protocol.

### Cell counting kit-8 (CCK-8) assay

The CCK-8 test (KeyGEN, Nanjing, China) was applied to evaluate the vitality of the cells. In 96-well plates, MLE-12 cells were planted with a 5 × 10^3^ cell density in each well. After adding CCK-8 reagent to each well after treatment, a microplate reader (Kehua Bio, Shanghai, China) was used to detect the absorbance at 450 nm.

### Evaluation of biochemical markers

The concentrations of glutathione (GSH), ferrous iron (Fe^2^⁺), and malondialdehyde (MDA) in MLE-12 cells were quantified using specific assay kits following the manufacturers’ protocols. Fe^2^⁺ concentrations were measured using an iron assay kit (Leagene, Beijing, China). The supernatant was obtained for investigation after lysing MLE-12 cells. The iron assay was performed according to the kit instructions, and absorbance was read at the specified wavelength with a microplate reader. MDA levels, indicative of lipid peroxidation, were ascertained by an MDA assay kit (Beyotime, Shanghai, China). Cell lysates were prepared and processed per the manufacturer’s protocol. The MDA reacts with thiobarbituric acid (TBA) to form a colored complex, which was measured spectrophotometrically. GSH levels were measured using a GSH assay kit (Beyotime, Shanghai, China), derived from the interaction of GSH with a chromogenic reagent, and absorbance was read at the specified wavelength.

### Measurement of mitochondrial reactive oxygen species (ROS)

The expression of mitochondrial reactive oxygen species (ROS) in MLE-12 cells were assessed utilizing the MitoSOX™ Red reagent (Invitrogen, Shanghai, China). MLE-12 cells were cultured in 24-well plates protected from light and exposed to 5 μM MitoSOX™ reagent solution for 10 minutes in a humidified atmosphere containing 5% CO_2_ at 37°C. After incubation, cells were rinsed twice with PBS to remove excess reagent. The fluorescence of MitoSOX™ Red, which specifically targets mitochondrial ROS, was visualized using a fluorescence microscope (Olympus, Shanghai, China). Images were captured under consistent settings to ensure comparability. The degree of mitochondrial ROS fluorescence in individual cells was measured with Image-Pro Plus software (Version 6.0).

### Quantitative real-time polymerase chain reaction (qRT-PCR)

The total RNA of MLE-12 cells was obtained utilizing the TRIzol reagent (Tiangen, Beijing, China) as directed by the manufacturer. For cDNA synthesis, we utilized a PrimeScript RT kit from Dalian, China. To do qRT-PCR with the StepOnePlus Real-Time PCR System (Applied Biosystems, Shanghai, China), SYBR Green PCR Master Mix (Takara, China) was utilized. The data were examined by the 2^-ΔΔCT^ approach, with GAPDH abundance serving as the standard. The amplification procedure made use of the primer sequences listed below: *FBLN1* forward: 5’- TGCAAGGCTGGCTTCTATTT-3’, *FBLN1* reverse: 5’- AGGGCTGTTGAGACACTCGT-3’. *TGF-β* forward: 5’- CGTCACTGGAGTTGTACGG-3’, *TGF-β* reverse: 5’-TGGTTGTAGAGGGCAAGGAC-3’. Similarly, the forward and reverse primers for GAPDH used as the reference gene, were as follows: *GAPDH* forward: 5′-GGAAAGCTGTGGCGTGATG-3′, *GAPDH* reverse: 5′-CTGTTGCTGTAGCCGTATTC-3′.

### Western blot (WB) assay

Protease and phosphatase inhibitors (CoWin Biosciences, Nanjing, China) were added to the RIPA lysis buffer (Solarbio, Beijing, China) to create protein lysates from MLE-12 cells. The BCA Protein Assay Kit (Beyotime, China) was utilized to ascertain the protein concentrations. On polyvinylidene difluoride (PVDF) membranes (Beyotime, Beijing, China), equal volumes of protein were separated by 10% SDS-PAGE. Membranes were blocked with 5% skim milk at room temperature for 1 hour and thereafter primary antibodies were incubated for an additional night at 4°C: FBLN1 (Abcam, China, 1:1000), GPX4 (Abcam, China, 1:1000), ACSL4 (Abcam, China, 1:1000), TGF-β (Abcam, China, 1:1000), p-Smad (Abcam, China, 1:1000), and SFXN1 (Proteintech Group, Inc, Wuhan, China, 1:1000). Subsequent washing, the membranes were treated for one hour at room temperature with HRP-conjugated secondary antibodies (Abcam, China, 1:5000). GAPDH (Abcam, China, 1:5000) was used as an internal reference. A ChemiDoc imaging system (Bio-Rad, Shanghai, China) was applied to acquire the images of the protein bands, which were viewed with an enhanced chemiluminescence (ECL) kit from Tiangen, Beijing, China. The original uncropped WB images are provided in S1 Raw images.

### Statistical analysis

The statistical study was carried out using the R program. Every experiment was conducted in triplicate, and the results were reported as mean ± SD. One-way ANOVA was utilized to evaluate the significance of differences, and Tukey’s test was utilized for post-hoc analysis. *P* less than 0.05 was employed as the statistical significance criterion.

## Results

### Identifying key modules in ALI using WGCNA

**Fig 1:**
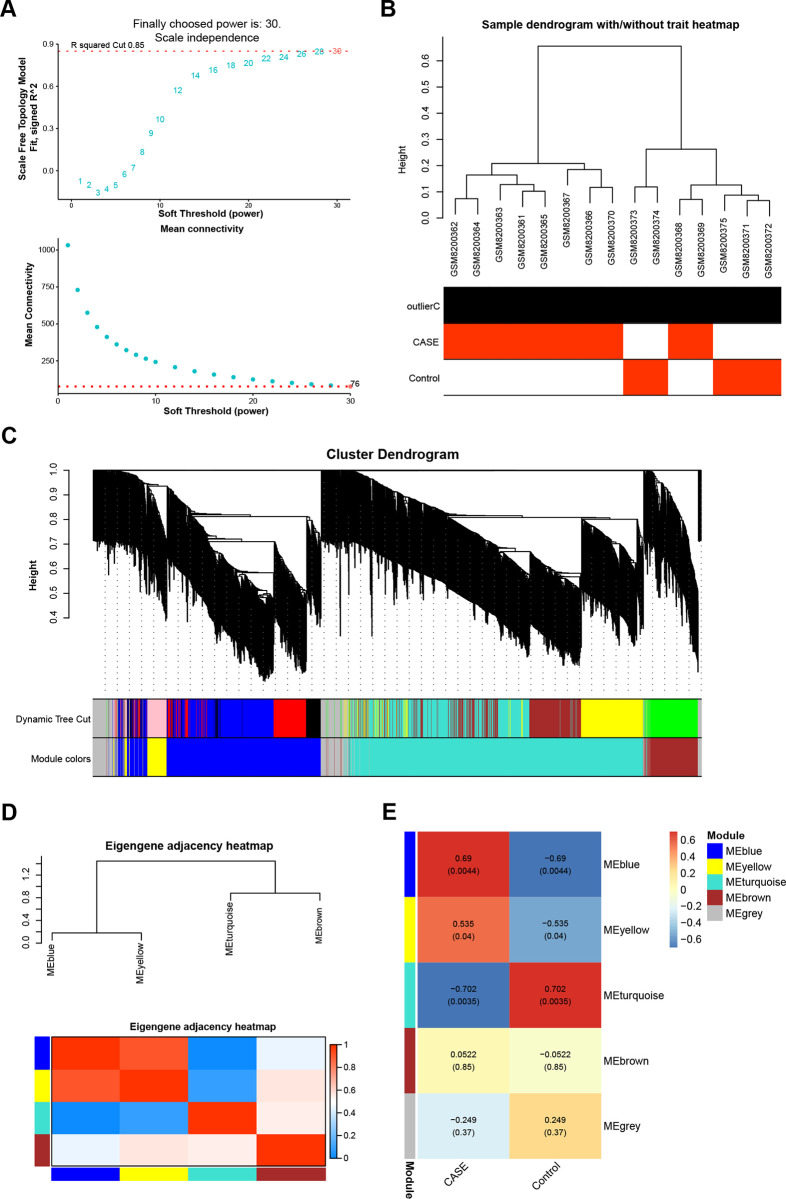
Gene co-expression network analysis of the GSE263867 dataset. (A) The upper figure shows the determination of the optimal soft threshold in the gene co-expression network, and the lower figure shows the mean connectivity for different soft thresholds. (B) Sample dendrogram and trait heat map, different branches represent different GSE263867 dataset samples. (C) Gene dendrogram obtained by average linkage hierarchical clustering. The colored rows below the dendrogram show module assignments determined by dynamic tree cuts. (D) Eigengene adjacency heat map of the correlation between module eigengenes and sample traits. (E) Heatmap of the correlation between gene modules and GSE263867 samples, the numbers in the modules represent the correlation coefficients and *p*-values.

To identify the most significant modules within the GSE263867 dataset, a WGCNA was performed on all genes (Fig 1A). Fig 1B depicts a scale-free topology with a scale independence of 0.85 when the soft threshold power was set to 30, along with high mean connectivity. Consequently, a power value of 30 was employed to construct the co-expression modules, resulting in the initial delineation of the modules. The WGCNA output illustrated that various modules were represented by distinct colors (Fig 1C). To find anomalies, a dendrogram was conducted utilizing the module eigengenes, followed by merging extremely close branches, with a merging threshold set at 0.5 (Fig 1D). A correlation analysis was then conducted between the eigengenes of each module and the sample traits to recognize the modules connected to particular phenotypes. The analysis revealed that genes within the MEturquoise module demonstrated a strong negative connection with ALI, with a -0.702 correlation coefficient (Fig 1E).

### Identification of key overlapping genes in ALI

**Fig 2:**
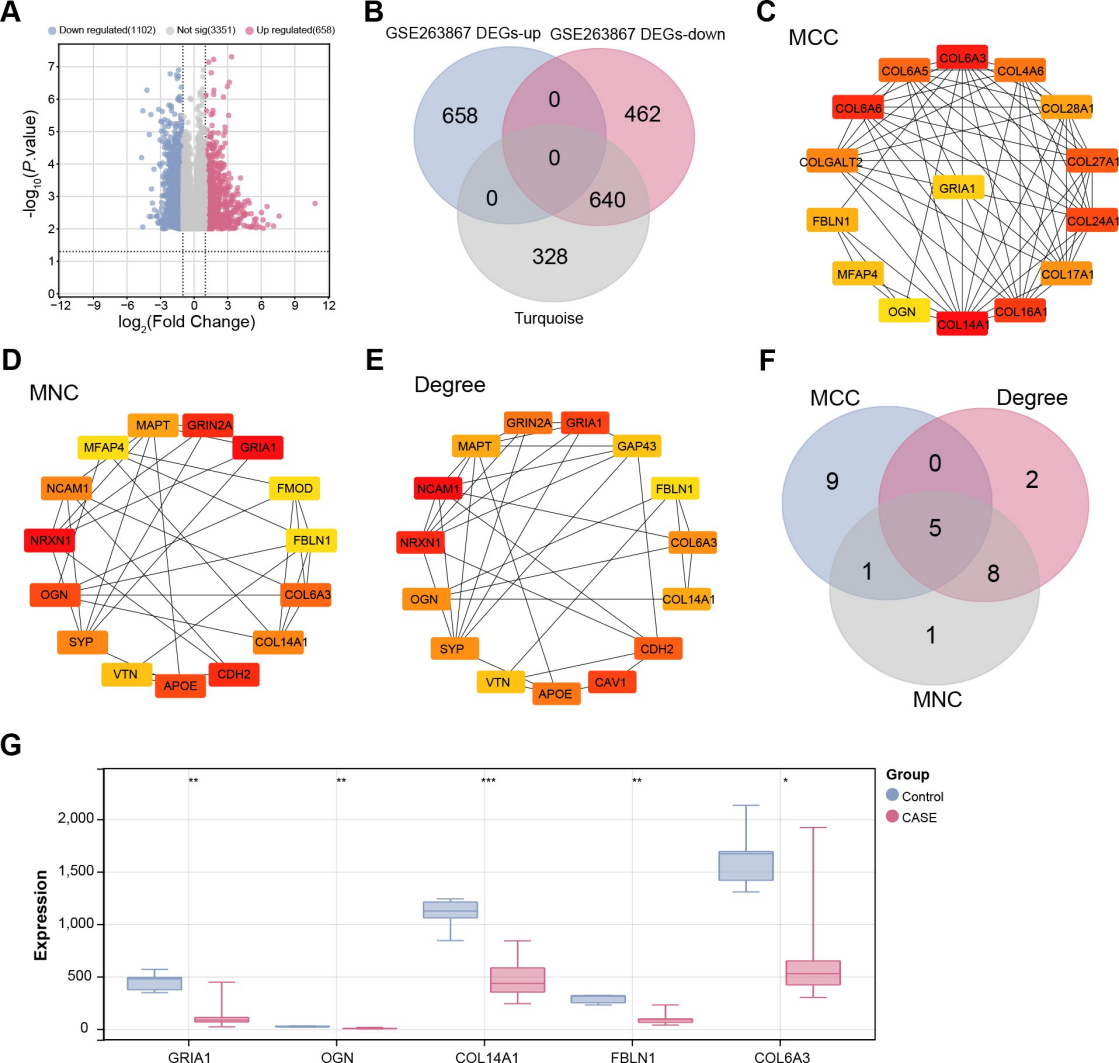
Identification of key overlapping genes in ALI. (A) Volcano plot depicting the results of differential gene expression analysis. Red dots represent up-regulated DEGs, and blue dots represent down-regulated DEGs. (B) Venn diagram of cross-analysis of up-regulated DEGs, down-regulated DEGs, and turquoise modules of the GSE263867 dataset to obtain overlapping genes. (C-E) PPI network analysis of overlapping genes, including MCC, MNC, and degree. Nodes represent proteins or protein domains, while edges represent interactions between these proteins. (F) This Venn diagram cross-analyzes three PPI modules, identifying five key overlapping genes. (G) Expression analysis of five key overlapping genes in control and case groups of the GSE263867 dataset. Red represents the case group, and blue represents the control group. ALI, Acute lung injury; DEGs, Differential Expressed Genes; PPI, protein-protein interactions; MCC, Maximal Clique Centrality; MNC, Maximal Neighborhood Component. *p<0.05, **p<0.01, ***p<0.001.

From the GSE263867 dataset, 1102 downregulated and 658 upregulated DEGs were detected between ALI samples and control samples, as illustrated in Fig 2A. Using bioinformatics tools, 640 common genes were identified among the upregulated DEGs, downregulated DEGs, and the turquoise module from the GSE263867 dataset (Fig 2B). A PPI network analysis of these 640 overlapping genes was performed, incorporating the MNC, MCC, and degree metrics (Fig 2C–2E). Subsequent cross-analysis of the genes within the three network modules using bioinformatics tools revealed five key overlapping genes: *GRIA1*, *OGN*, *COL14A1*, *FBLN1*, and *COL6A3* (Fig 2F). Expression analysis demonstrated that these five key overlapping genes were significantly downregulated in the ALI samples from the GSE263867 dataset (Fig 2G).

### LPS induces ferroptosis of lung epithelial cells

**Fig 3:**
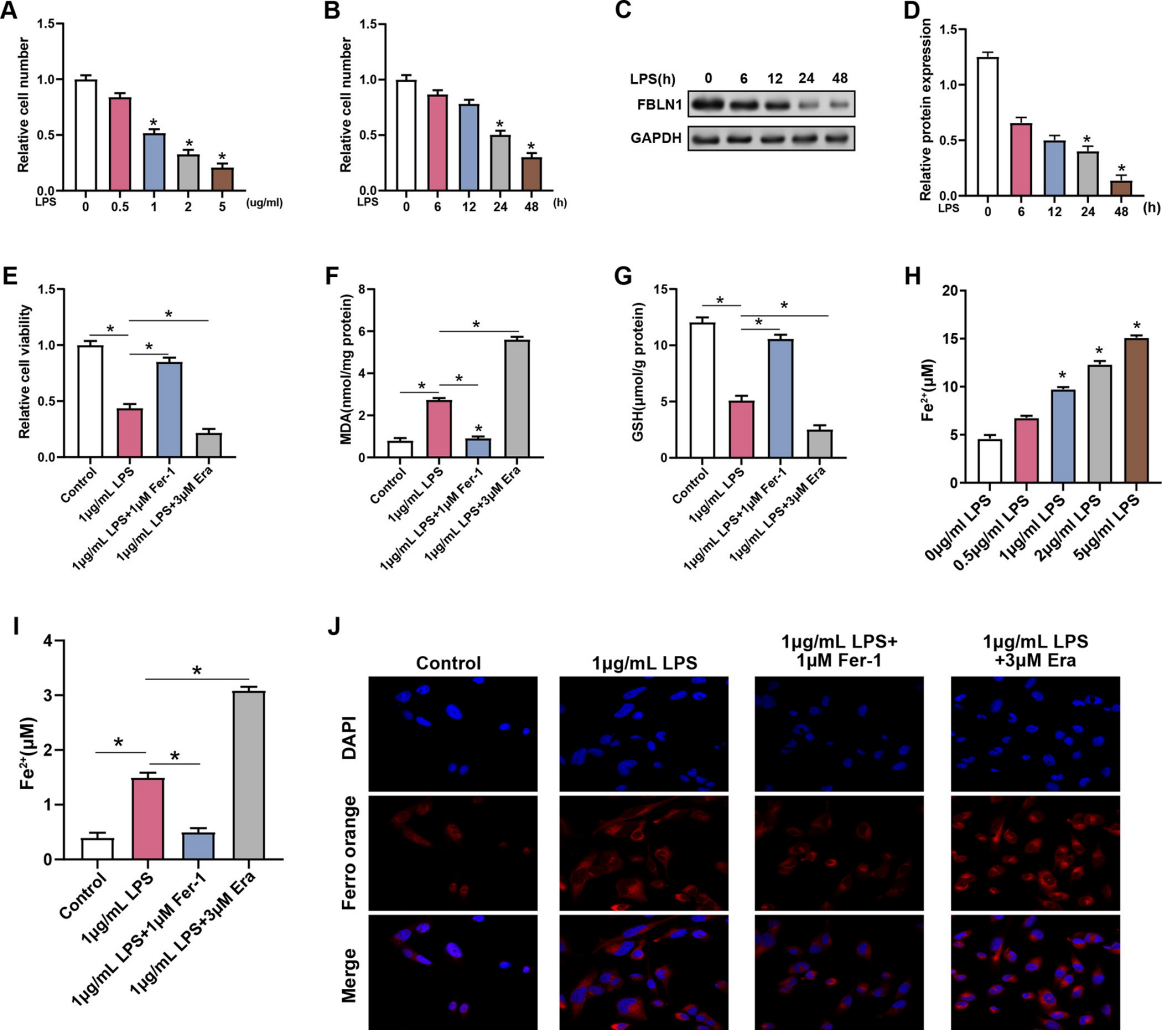
LPS induces ferroptosis of lung epithelial cells. (A) CCK-8 assay to detect the cell number of MLE-12 cells treated with different concentrations of LPS (0.5, 1, 2, 5μg/mL) for 24 hours. (B) CCK-8 assay to detect the cell number of MLE-12 cells treated with 1μg/mL LPS for 6, 12, 24, and 48 hours. (C and D) WB assay to detect the protein level of FBLN1 in MLE-12 cells treated with 1μg/mL LPS for 6, 12, 24, and 48 hours. (E) Relative cell viability of the control, LPS (1 μg/mL), LPS (1 μg/mL) + Ferrostatin-1 (1 μM), and LPS (1 μg/mL) + Erastin (3 μM) treatment groups was measured by CCK-8 assay. (F) MDA kit assay to detect the effect of LPS (1 μg/mL), LPS+Ferrostatin-1 (1μM) and LPS+Erastin (3μM) on MDA concentration in MLE-12 cells. (G) A GSH kit was used to detect the effect of LPS (1 μg/mL), LPS+Ferrostatin-1 (1μM) and LPS+Erastin (3μM) on GSH concentration in MLE-12 cells. (H) The Fe^2^⁺ assay kit was used to detect the effect of different concentrations of LPS (0.5 μg/mL, 1 μg/mL, 2 μg/mL, and 5 μg/mL) on Fe^2^⁺ concentration in MLE-12 cells. (I) Fe^2+^ kit was used to detect the effect of LPS (1 μg/mL), LPS+Ferrostatin-1 (1μM) and LPS+Erastin (3μM) on Fe^2+^ concentration in MLE-12 cells. CCK-8, Cell counting kit-8; LPS, lipopolysaccharide; WB, Western blot; MDA, malondialdehyde; GSH, glutathione; Fe^2+^, ferrous iron. (J) FerroOrange probes were used to detect the impact of LPS (1 μg/mL), LPS+Ferrostatin-1 (1 μM), and LPS+Erastin (3 μM) on Fe2+ levels in MLE-12 cells. *p<0.05.

The relative number of MLE-12 cells administered with different LPS doses (0.5, 1, 2, 5 μg/mL) for 24 hours was assessed using the CCK-8 assay. Results revealed a considerable decrease in cell viability with increasing LPS concentrations (Fig 3A). Additionally, MLE-12 cells exposed for 6, 12, 24, and 48 hours to 1 μg/mL LPS exhibited a time-dependent reduction in cell viability, particularly notable at 12 and 48 hours (Fig 3B). For subsequent experiments, cells were subjected to 1 μg/mL LPS for 24 hours. WB analysis revealed that FBLN1 protein levels in MLE-12 cells significantly decreased over time following treatment with 1 μg/mL LPS (Fig 3C and 3D). To learn more about the function of ferroptosis in this process, the ferroptosis inhibitor Ferrostatin-1 (Fer-1) and the ferroptosis inducer Erastin (Era) were applied to the cells. CCK-8 and GSH assays indicated that Fer-1 eased the LPS-induced reduction in cell viability and GSH levels, whereas Era exacerbated these reductions (Fig 3E and 3G). Moreover, measurements of MDA and Fe^2^⁺ concentrations demonstrated that LPS treatment significantly increased these oxidative stress markers in MLE-12 cells. The addition of Fer-1 counteracted the LPS-induced increases in MDA and Fe^2^⁺ levels, while Era intensified these effects (Fig 3F and 3I). Furthermore, we measured the changes in the concentration of the ferroptosis marker Fe^2^⁺ after adding varying concentrations of LPS. Our results demonstrated that as the concentration of LPS increased, the Fe^2^⁺ concentration also increased gradually. This indicates a correlation between the changes in Fe^2^⁺ levels and the concentration of LPS, thereby confirming that the induction of ferroptosis is indeed concentration-dependent. FerroOrange staining revealed that compared to the LPS-treated group, Fer-1 mitigated the LPS-induced increase in Fe2+ levels, while Era exacerbated these effects (Fig 3J). These findings suggest that LPS induces ferroptosis in MLE-12 cells, characterized by reduced cell viability and GSH levels, increased lipid peroxidation, and elevated Fe^2^⁺ levels. Fer-1 mitigated these effects, highlighting its capacity as an agent of protection against LPS-induced ferroptosis, whereas Erastin exacerbated these effects, confirming its role as a ferroptosis inducer.

### Overexpression of *FBLN1* inhibits ferroptosis in LPS-induced ALI

**Fig 4:**
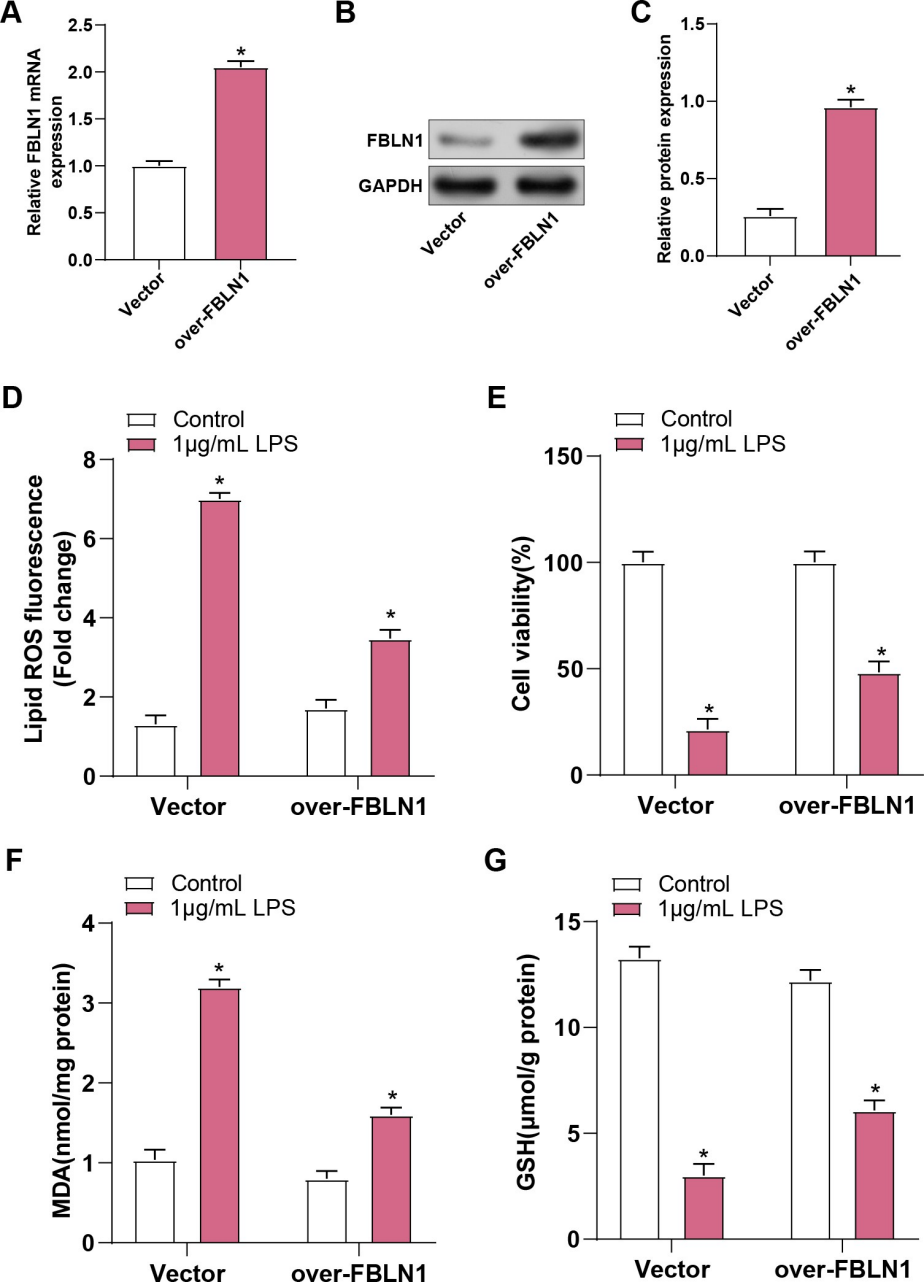
Overexpression of *FBLN1* can inhibit ferroptosis in LPS-induced ALI. (A-C) qRT-PCR and WB were used to detect the overexpression efficiency of *FBLN1* in MLE-12 cells. (D) An ROS kit was used to detect the lipid ROS level of MLE-12 cells after 1 μg/mL LPS+over-*FBLN1* treatment. (E) CCK-8 was used to detect the changes in cell viability of MLE-12 cells after 1 μg/mL LPS+over-*FBLN1* treatment. (F) An MDA kit was used to detect the changes in the MDA concentration of MLE-12 cells after 1 μg/mL LPS+over-*FBLN1* treatment. (G) A GSH kit was used to detect the changes in the GSH concentration of MLE-12 cells after 1 μg/mL LPS+over-*FBLN1* treatment. ALI, Acute lung injury; CCK-8, Cell counting kit-8; LPS, lipopolysaccharide; qRT-PCR, Quantitative real-time polymerase chain reaction; WB, Western blot; MDA, malondialdehyde; GSH, glutathione; ROS, reactive oxygen species. *p<0.05.

The efficiency of *FBLN1* overexpression in MLE-12 cells was verified by qRT-PCR and WB analyses, demonstrating substantial rises in *FBLN1* mRNA and protein levels (Fig 4A–4C). Consistent with previous findings, LPS-treated cells showed significant accumulation of lipid ROS. However, *FBLN1* overexpression partially reversed these effects, resulting in reduced lipid ROS levels (Fig 4D). LPS treatment resulted in significant decreases in cell viability, and *FBLN1* overexpression alleviated these decreases and partially restored cell viability (Fig 4E). In addition, *FBLN1* overexpression partially reversed the significant accumulation of MDA caused by LPS, resulting in reduced MDA levels (Fig 4F). LPS treatment also caused a significant decrease in GSH levels, and *FBLN1* overexpression partially restored GSH levels (Fig 4G). These outcomes suggest that *FBLN1* has a protective function in counteracting LPS-induced oxidative stress and maintaining cell viability. To further investigate the role of FBLN1, we employed siRNA to knock down FBLN1 expression and evaluated the effects on lipid ROS, MDA, GSH, and Fe^2^⁺ levels in MLE-12 cells treated with 1 μg/mL LPS. The results demonstrated that FBLN1 knockdown enhanced LPS-induced increases in lipid ROS, MDA, and Fe^2^⁺ levels, while simultaneously inhibiting the reduction of GSH levels induced by LPS (S1 Fig). These findings are contrary to those observed with FBLN1 overexpression and further underscore the protective role of FBLN1 against LPS-induced oxidative stress.

### FBLN1 inhibits ferroptosis by reducing intracellular free ferrous iron

**Fig 5:**
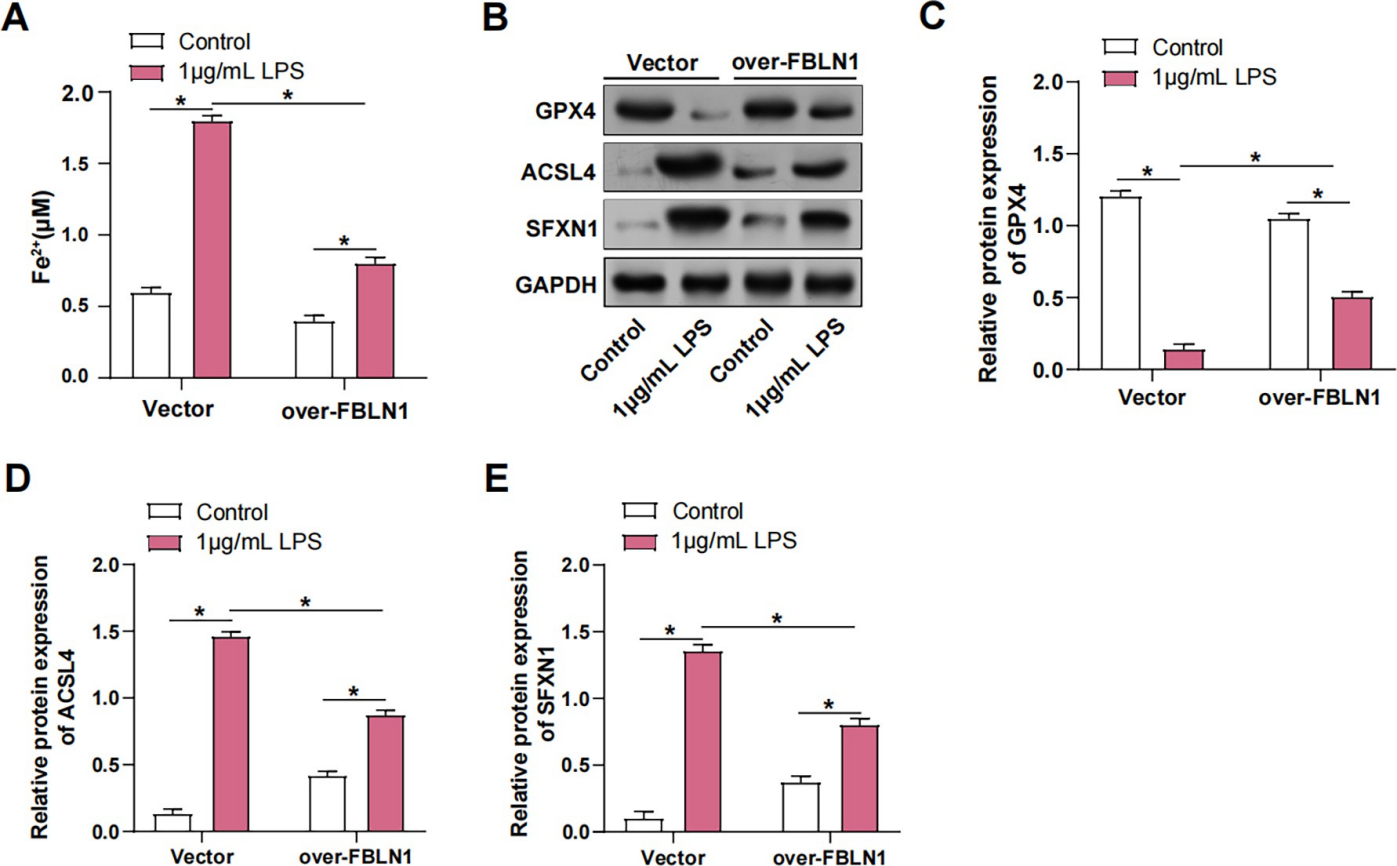
FBLN1 inhibits ferroptosis by reducing intracellular free ferrous iron. (A) Fe^2+^ kit was used to detect the changes in Fe^2+^ concentration in MLE-12 cells after 1 μg/mL LPS+over-FBLN1 treatment. (B-E) WB was used to detect the changes in GPX4, ACSL4, and SFXN1 protein levels in MLE-12 cells after 1 μg/mL LPS+over-FBLN1 treatment. LPS, lipopolysaccharide; WB, Western blot; Fe^2+^, ferrous iron. *p<0.05.

Following LPS treatment, a significant increase in Fe^2^⁺ levels was observed. However, overexpression of FBLN1 in MLE-12 cells reduced the extent of Fe^2^⁺ accumulation (Fig 5A). The expression of GPX4, a critical regulator of ferroptosis, was suppressed following LPS stimulation. Notably, the overexpression of FBLN1 alleviated the LPS-induced suppression of GPX4 expression (Fig 5B). Furthermore, LPS stimulation promoted the expression of ACSL4 and SFXN1, both of which are associated with ferroptosis. Overexpression of FBLN1 partially reversed the LPS-induced upregulation of ACSL4 and SFXN1. These results suggest that FBLN1 overexpression mitigates LPS-induced ferroptosis by modulating Fe^2^⁺ accumulation and the levels of essential proteins that participated in the ferroptosis pathway.

### FBLN1 regulates ferroptosis by inhibiting TGF-β

**Fig 6:**
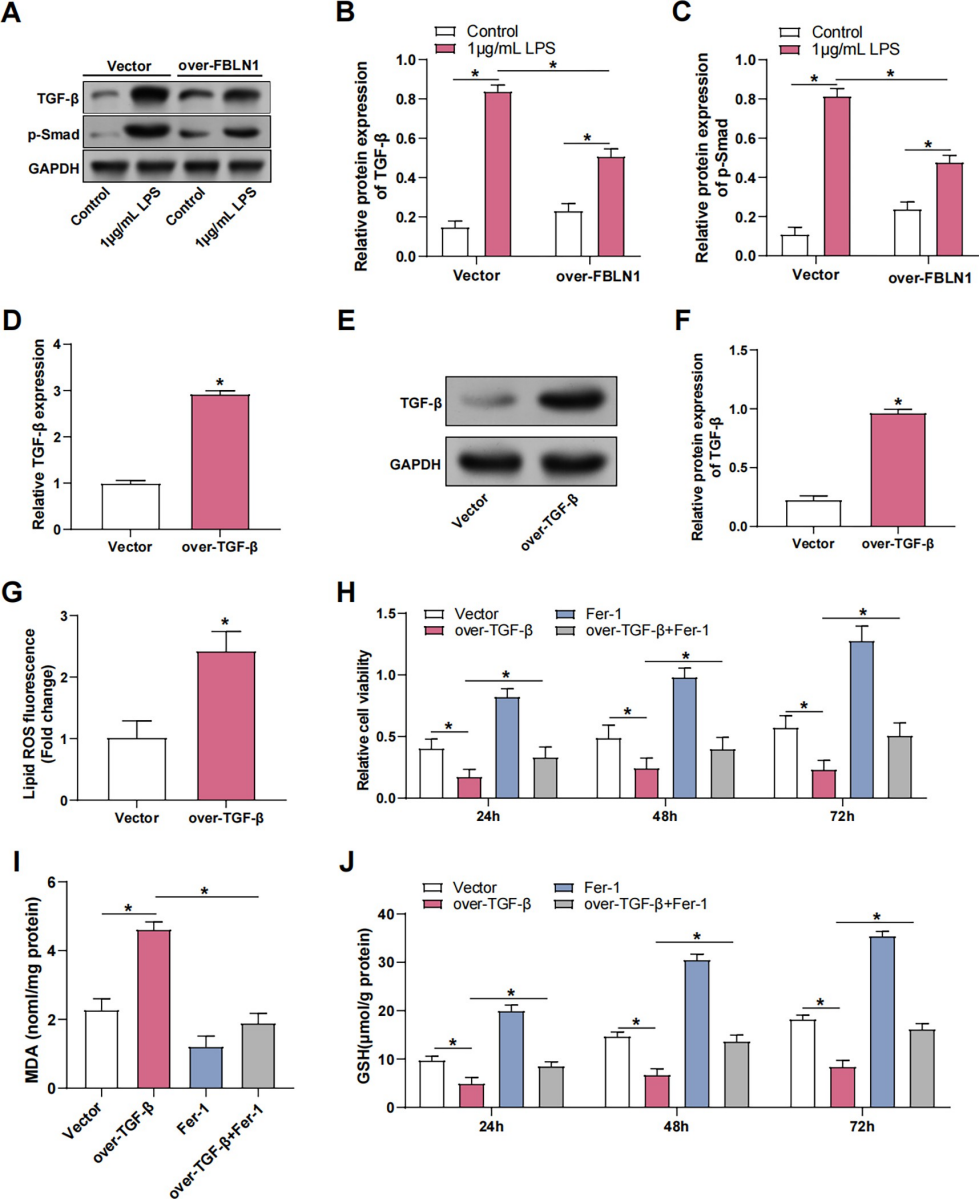
FBLN1 regulates ferroptosis by inhibiting TGF-β. (A-C) WB detection of TGF-β and p-Smad protein levels in MLE-12 cells after 1 μg/mL LPS + over-FBLN1 treatment. (D-F) qRT-PCR and WB were used to detect the efficiency of TGF-β overexpression in MLE-12 cells treated with 1 μg/mL LPS. (G) The lipid ROS level was detected by ROS kit after overexpression of TGF-β in MLE-12 cells treated with 1 μg/mL LPS. (H) CCK-8 was used to detect the changes in the viability of MLE-12 cells treated with 1 μg/mL LPS. The groups were: Vehicle, excessive TGF-β, Fer-1, and excessive TGF-β + Fer-1. (I) The changes in MDA concentration in MLE-12 cells treated with 1 μg/mL LPS were detected by MDA kit. The groups were vehicle, excess TGF-β, Fer-1, excess TGF-β+Fer-1. (J) GSH kit was used to detect the changes in GSH concentration in MLE-12 cells treated with 1 μg/mL LPS. The groups were vehicle, excess TGF-β, Fer-1, and excess TGF-β+Fer-1. CCK-8, Cell counting kit-8; LPS, lipopolysaccharide; qRT-PCR, Quantitative real-time polymerase chain reaction; WB, Western blot; MDA, malondialdehyde; GSH, glutathione; ROS, reactive oxygen species; Fer-1, Ferrostatin-1. *p<0.05.

WB analysis showed that treatment of MLE-12 cells with 1 μg/mL LPS for 24 h markedly enhanced TGF-β and p-Smad protein levels. FBLN1 overexpression alleviated this effect (Fig 6A–6C). qRT-PCR and WB confirmed the efficiency of TGF-β overexpression in MLE-12 cells, showing that TGF-β was highly upregulated at the levels of proteins and mRNA (Fig 6D–6F). ROS assay showed that TGF-β overexpression significantly increased lipid ROS levels (Fig 6G). Further experimental results showed that with the increase in the duration of TGF-β overexpression (24, 48, 72 h), cell viability (Fig 6H) and GSH levels gradually increased (Fig 6J), while MDA levels were significantly increased (Fig 6I). However, the addition of Ferrostatin-1 reversed these changes and restored cell viability, GSH levels, and MDA levels. These results suggest that TGF-β overexpression exacerbates LPS-induced ferroptosis and oxidative stress in MLE-12 cells, whereas Ferrostatin-1 treatment alleviates these effects.

### FBLN1 regulates ferroptosis by reducing free ferrous iron through inhibiting TGF-β/Smad pathway

**Fig 7:**
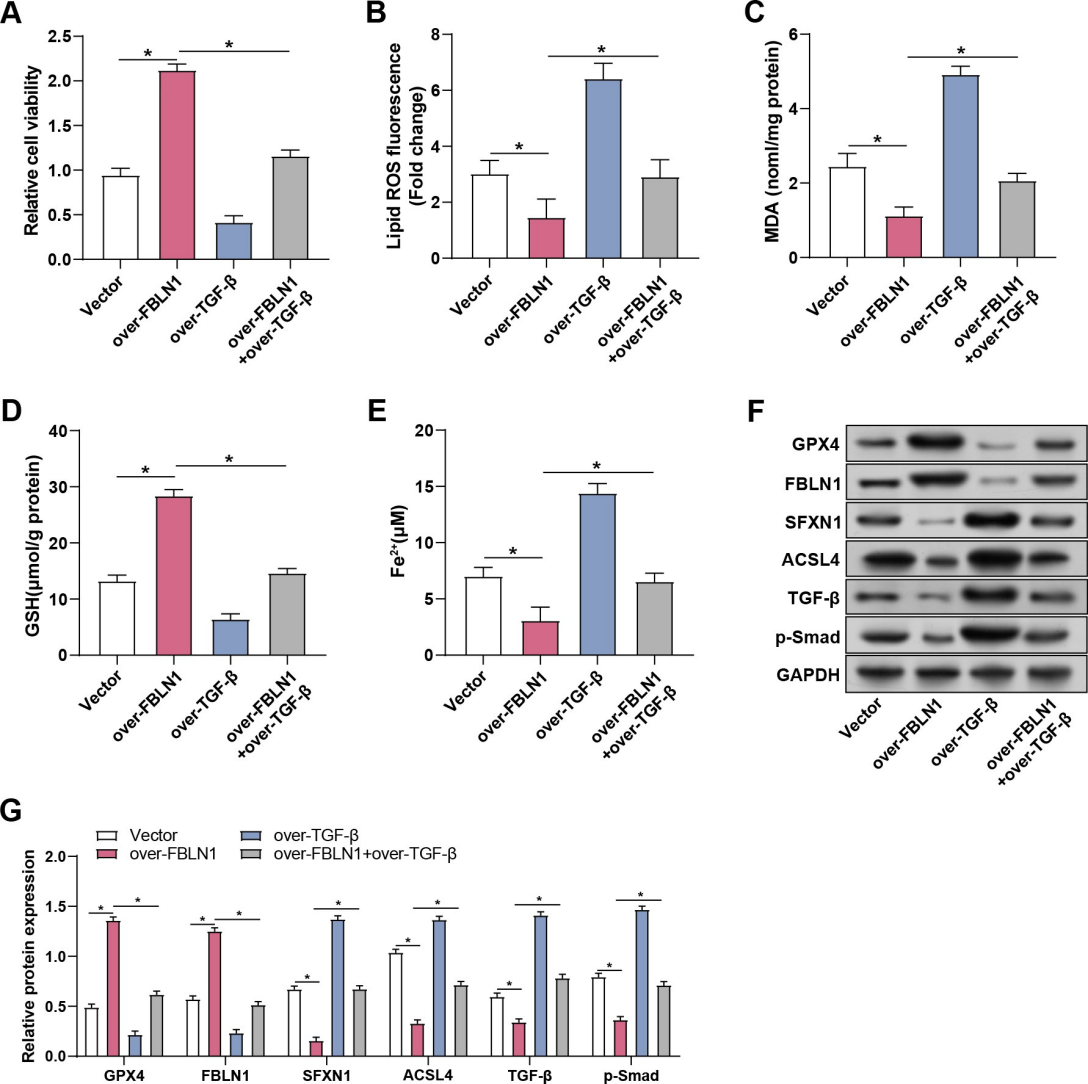
FBLN1 regulates ferroptosis by reducing free ferrous iron by inhibiting TGF-β/Smad pathway. (A-E) Changes in cell viability, lipid ROS level, MDA concentration, GSH concentration, and Fe^2+^ concentration in MLE-12 cells treated with 1 μg/mL LPS were detected. The groups were: Vehicle, FBLN1 overexpression, TGF-β overexpression, and FBLN1 overexpression + TGF-β overexpression. (F and G) WB was used to detect the protein levels of GPX4, FBLN1, SFXN1, ACSL4, TGF-β, and p-Smad in MLE-12 cells treated with 1 μg/mL LPS. CCK-8, Cell counting kit-8; LPS, lipopolysaccharide; WB, Western blot; MDA, malondialdehyde; GSH, glutathione; ROS, reactive oxygen species; Fe^2+^, ferrous iron. *p<0.05.

After 1 μg/mL LPS was applied to the MLE-12 cells for 24 hours, CCK-8, ROS, MDA, GSH, and Fe^2+^ assays were used to evaluate. The results showed that overexpression of FBLN1 significantly reduced cellular lipid ROS, MDA, and Fe^2+^ levels, while significantly increasing cell viability and GSH levels. However, these effects were counteracted by overexpression of TGF-β (Fig 7A–7E). WB analysis showed that overexpression of *FBLN1* significantly increased the protein expression of *FBLN1* and *GPX4*, while the protein levels of *SFXN1*, *ACSL4*, *TGF-β*, and p-Smad were significantly decreased. Overexpression of *TGF-β* reversed these changes, returning expression levels to those observed when treated with LPS alone (Fig 7F and 7G). These findings suggest that FBLN1 overexpression alleviates LPS-induced ferroptosis and oxidative stress in MLE-12 cells by regulating key proteins involved in these pathways. However, TGF-β overexpression counteracts these protective effects, suggesting a role in promoting oxidative stress and ferroptosis.

## Discussion

In this study, we performed WGCNA on the entire gene set of the GSE263867 dataset, identifying the turquoise module as a key module of interest. Through intersectional analysis with upregulated and downregulated genes in this dataset, we identified common genes and subsequently conducted a PPI network analysis. Cross-referencing genes from three PPI modules revealed five crucial overlapping genes: *GRIA1*, *OGN*, *COL14A1*, *FBLN1*, and *COL6A3*. Expression analysis of these genes in the GSE263867 dataset samples was conducted. Prior research by Zhang Y et al. has demonstrated the involvement of *GRIA1* in the pathogenesis of ARDS through miRNA-mRNA regulatory networks, validating its role in a bleomycin-induced ALI mouse model [13]. Additionally, Shi S et al. have shown that miR-140 overexpression attenuates pulmonary fibrosis in interstitial lung disease (ILD) by downregulating *OGN* and activating the Wnt signaling pathway, which affects lung fibroblast apoptosis, proliferation, and fibrosis-related gene expression [14]. These studies collectively emphasize the intricate molecular mechanisms underlying ARDS, identifying potential therapeutic targets. Given the limited research on *FBLN1* in ARDS, we selected it as a hub gene for further investigation.

LPS is a main part of the outer membrane of Gram-negative bacteria and is renowned for its potent ability to elicit strong immune responses in mammals [15]. Due to its robust pro-inflammatory properties, LPS is commonly used to induce inflammation in various cell types, including MLE-12 cells, which are murine lung epithelial cells [16]. This model helps researchers study the cellular mechanisms of inflammation and identify potential therapeutic targets [17]. Inflammation is an intricate biological reaction to detrimental influences, including damaged cells, pathogens, or irritants, and is characterized by the activation of immune cells, release of cytokines, and production of ROS [18]. In the context of LPS-induced inflammation, the study of therapeutic agents like Fer-1 and Era is crucial. These agents have been shown to mitigate inflammatory responses, thereby protecting cells from damage [19]. Furthermore, oxidative stress plays an important role in inflammation, which is characterized by an imbalance between the antioxidant defense system and ROS generation, including a decrease in MDA and GSH [20]. In LPS-induced ALI, oxidative stress and inflammation are prominent. Studies reveal various therapeutic targets and treatments for mitigating these effects. Zou G et al. found that miR-135a-5p suppresses *TBK1*, reducing pro-inflammatory cytokines and oxidative stress markers while activating the *NRF2/TXNIP* antioxidant pathway [21]. Similarly, Zimmermann KK et al. demonstrated that hydrogen sulfide (H_2_S) inhibits pro-inflammatory cytokines and reduces oxidative stress markers, such as *NADPH oxidase 2* and ROS, thereby preventing lung damage [22]. Additionally, Zhu W et al. showed that Schisandrin B (Sch B) directly targets *MyD88*, inhibiting key inflammatory pathways, reducing inflammatory cytokine production, and preventing oxidative stress, highlighting its therapeutic potential for ALI [23]. Our study found that LPS induced ferroptosis in lung epithelial cells, reduced survival rate, and increased oxidative stress markers, which were inhibited by Fer-1 and aggravated by Era. Overexpression of *FBLN1* reversed the damage caused by LPS and restored cell survival rate and GSH level. This suggests that *FBLN1* has a protective role in counteracting LPS-induced oxidative stress and maintaining cell survival.

Ferroptosis is a type of controlled cell death, different from apoptosis, necrosis, and autophagy in that it is characterized by iron-dependent lipid peroxidation [24]. It is driven by the buildup of lethal lipid ROS, which leads to cell membrane damage [25]. In ALI, ferroptosis contributes to tissue damage and inflammation, exacerbating the condition [26]. Elevated iron levels and oxidative stress in ALI promote ferroptosis, leading to increased cell death and impaired lung function. Zou X et al. found that *STEAP1* expression is elevated in sepsis-induced ALI models, and its inhibition reduces inflammation, ROS production, and MDA levels while increasing GSH levels, cell viability, and restoring mitochondrial morphology by affecting the *SLC7A11/GPX4* axis [27]. Similarly, Zhu Z et al. demonstrated that the SP-NK1R signaling pathway exacerbates inflammation and ferroptosis in the liver and lungs in a mouse model of sepsis brought on by surgery involving a cecal ligation puncture (CLP) [28]. Suppression of SP-NK1R signaling through *Tac1* gene deletion or NK1R blockade significantly reduced inflammatory responses, ferroptosis markers, and tissue injury. Zhou H et al. found that ferroptosis contributes to ALI induced by oleic acid (OA) in mice. This relationship is marked by iron overload, GSH depletion, MDA accumulation, and down-regulation of *GPX4* and ferritin [29]. Thus, targeting *STEAP1* and SP-NK1R signaling pathways, along with addressing ferroptosis, may provide therapeutic benefits in mitigating sepsis-induced ALI by reducing ferroptosis and associated inflammation. Our study found that Fe^2+^ levels were significantly increased after LPS treatment, but overexpression of FBLN1 in MLE-12 cells reduced Fe^2+^ accumulation. FBLN1 overexpression reversed LPS-induced upregulation of ACSL4 and SFXN1. These findings indicated that FBLN1 overexpression reduces LPS-induced ferroptosis by controlling Fe^2^⁺ accumulation and the levels of key proteins connected to the ferroptosis pathway. Comprehending the function of ferroptosis in ALI offers new perspectives on potential therapeutic targets aimed at alleviating lung injury by inhibiting the ferroptosis pathway.

The TGF-β/Smad pathway is an important cascade of signals that controls a number of biological functions, including as apoptosis, differentiation, and proliferation [30]. When *TGF-β* ligands bind to their receptors, they induce the phosphorylation of receptor-regulated Smad proteins (R-Smads), which then combine with common-mediator Smads (Co-Smads) to create complexes [31]. To control the expression of the target genes, these complexes are transferred to the nucleus. This pathway has a crucial function in the modulation of inflammation and tissue fibrosis. In the context of inflammation, *TGF-β* signaling has the ability to have both pro- and anti-inflammatory actions, depending on the cellular environment and context [32]. It promotes the resolution of inflammation by inducing anti-inflammatory cytokines and inhibiting pro-inflammatory responses. Bao R et al. highlight its role in exacerbating silicosis through fibrosis and ferroptosis, proposing inhibition of this pathway alongside HGF therapy as a promising treatment strategy [33]. Yao Y et al. emphasize its contribution to pulmonary fibrosis via inflammation, oxidation, and collagen deposition, alleviated by Nervilia fordii extract through modulation of a-SMA, *TGF-β1*, *CTGF*, *Smad3/4*, *Smad7*, and ERK1/2 [34]. Wang L et al. further elucidate its involvement in ARDS-associated lung fibrosis, exacerbated by miR-425 reduction-induced *Smad2* expression via histone demethylation, highlighting the role of pathways across diverse lung fibrotic conditions and potential therapeutic avenues [35]. Our study showed that *TGF-β* overexpression exacerbated LPS-induced oxidative stress, with Fer-1 indicating the involvement of the ferroptosis pathway. *FBLN1* attenuated oxidative stress and ferroptosis by modulating TGF-β/Smad pathway proteins. Conversely, *TGF-β* overexpression reversed these protective effects, implicating its role in promoting oxidative stress and ferroptosis. Targeting the TGF-β/Smad pathway may thus hold therapeutic promise for ALI and other inflammatory conditions driven by inflammation and ferroptosis.

While our in vitro findings provide valuable insights into the role of *FBLN1* in regulating ferroptosis in ALI, we acknowledge that the absence of in vivo validation is a limitation of this study. The cellular experiments, although robust, may not fully recapitulate the complex physiological environment and disease pathology present in a whole organism. The interactions between different cell types, the immune system, and the intricate interplay of signaling pathways in vivo could potentially modulate the effects of *FBLN1* in ways that are not captured by our current experiments. Future studies will need to address these considerations by extending our investigations to animal models of ALI. Such in *vivo* experiments will be crucial for validating the therapeutic potential of *FBLN1* modulation and for elucidating the broader implications of our findings in the context of whole-body physiology and disease progression. By integrating in vivo data with our current in *vitro* results, a more comprehensive understanding of *FBLN1*’s role in ALI can be achieved, ultimately facilitating the development of targeted therapies for this devastating condition.

## Conclusion

Through WGCNA analysis of the GSE263867 dataset, this study identified important gene modules associated with ARDS, among which the turquoise module stood out due to its negative correlation with ARDS. Integration of differential gene expression pro S2s and PPI networks highlighted key genes, including *GRIA1*, *OGN*, *COL14A1*, *FBLN1*, and *COL6A3*, which are key players in the pathogenesis of ARDS. Studies on LPS-induced ferroptosis in MLE-12 cells showed that *FBLN1* overexpression alleviated oxidative stress and maintained cell viability by reducing ROS, MDA, and Fe^2^⁺ levels. This protective effect was mediated by upregulating GPX4 and downregulating ACSL4 and SFXN1. In contrast, TGF-β overexpression antagonized these beneficial effects, exacerbated oxidative stress, and promoted ferroptosis. These findings highlight the therapeutic potential of targeting the TGF-β/Smad pathway and enhancing *FBLN1* expression to alleviate ARDS-associated oxidative stress and ferroptosis. This study provides new insights into therapeutic strategies for inflammatory lung diseases, highlighting *FBLN1* as a promising therapeutic target to prevent ARDS via controlling important proteins that participate in oxidative stress and ferroptosis pathways.

## Supporting information

S1 Fig Knockdown of FBLN1 can promote ferroptosis in LPS-induced ALI. (A) qRT-PCR was used to detect the knockdown efficiency of *FBLN1* in MLE-12 cells. (B) WB was used to detect the knockdown efficiency of *FBLN1* in MLE-12 cells. (C) An ROS kit was used to detect the lipid ROS level of MLE-12 cells after 1 μg/mL LPS+si-*FBLN1* treatment. (D) An MDA kit was used to detect the changes in the MDA concentration of MLE-12 cells after 1 μg/mL LPS+si-*FBLN1* treatment. (E) A GSH kit was used to detect the changes in the GSH concentration of MLE-12 cells after 1 μg/mL LPS+si-*FBLN1* treatment. (F) Fe^2+^ kit was used to detect the changes in the Fe^2+^ concentration of MLE-12 cells after 1 μg/mL LPS+si-*FBLN1* treatment. ALI, Acute lung injury; CCK-8, Cell counting kit-8; LPS, lipopolysaccharide; qRT-PCR, Quantitative real-time polymerase chain reaction; WB, Western blot; MDA, malondialdehyde; GSH, glutathione; ROS, reactive oxygen species. *p<0.05.
(TIF)

S1 Raw images Original uncropped Western blot images. (TIF)
