# Supplementary figures and images for: FBLN1 regulates ferroptosis in acute respiratory distress syndrome by reducing free ferrous iron by inhibiting the TGF-β/Smad pathway
Source: PLoS One. 2024 Dec 13;19(12):e0314750. doi: 10.1371/journal.pone.0314750 (PMC11643259; doi:10.1371/journal.pone.0314750)

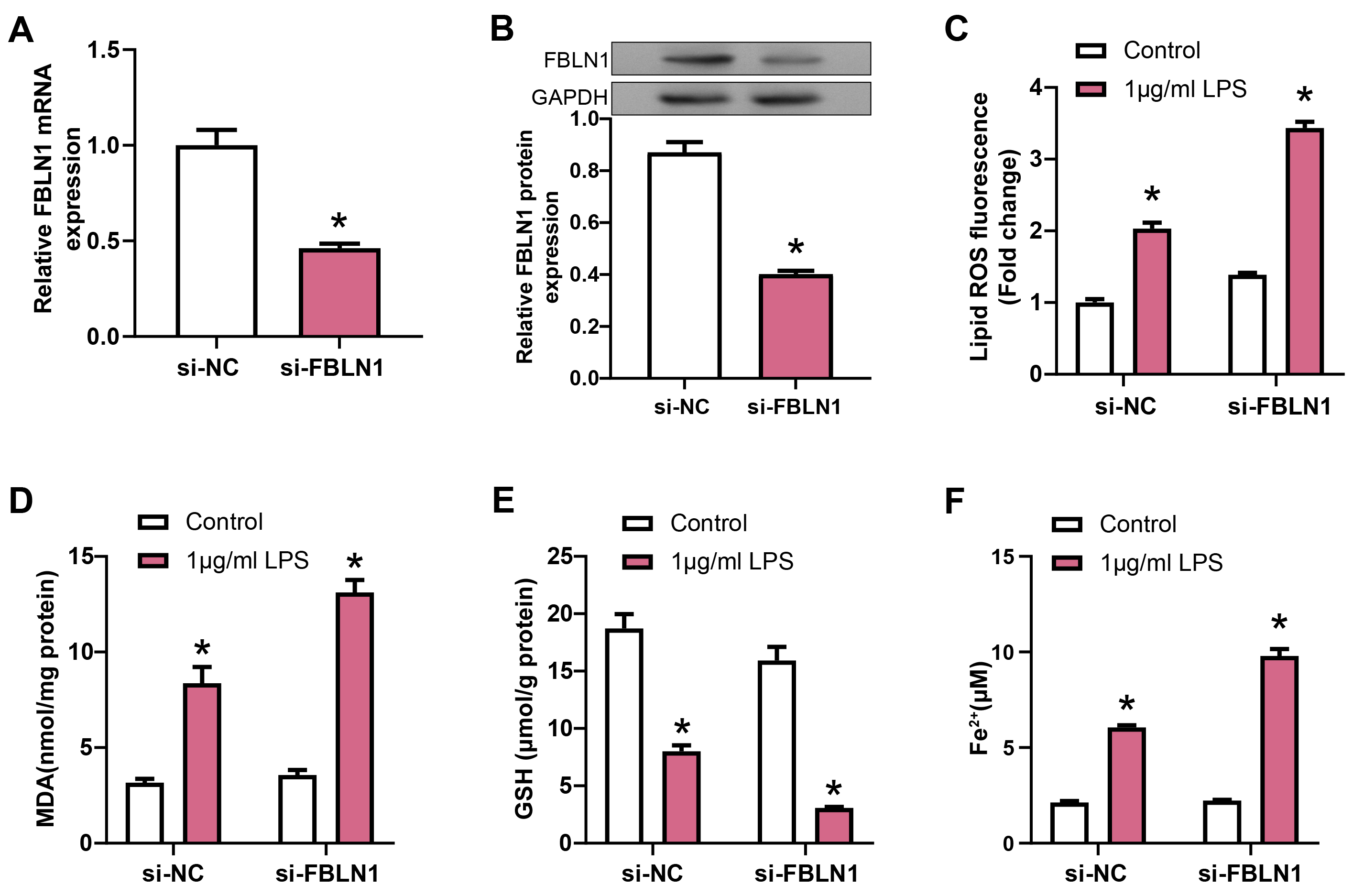

Supplement: S1 Fig — (A) qRT-PCR was used to detect the knockdown efficiency of FBLN1 in MLE-12 cells. (B) WB was used to detect the knockdown efficiency of FBLN1 in MLE-12 cells. (C) An ROS kit was used to detect the lipid ROS level of MLE-12 cells after 1 μg/mL LPS+si-FBLN1 treatment. (D) An MDA kit was used to detect the changes in the MDA concentration of MLE-12 cells after 1 μg/mL LPS+si-FBLN1 treatment. (E) A GSH kit was used to detect the changes in the GSH concentration of MLE-12 cells after 1 μg/mL LPS+si-FBLN1 treatment. (F) Fe2+ kit was used to detect the changes in the Fe2+ concentration of MLE-12 cells after 1 μg/mL LPS+si-FBLN1 treatment. ALI, Acute lung injury; CCK-8, Cell counting kit-8; LPS, lipopolysaccharide; qRT-PCR, Quantitative real-time polymerase chain reaction; WB, Western blot; MDA, malondialdehyde; GSH, glutathione; ROS, reactive oxygen species. *p<0.05. (TIF) [file pone.0314750.s001.tif]

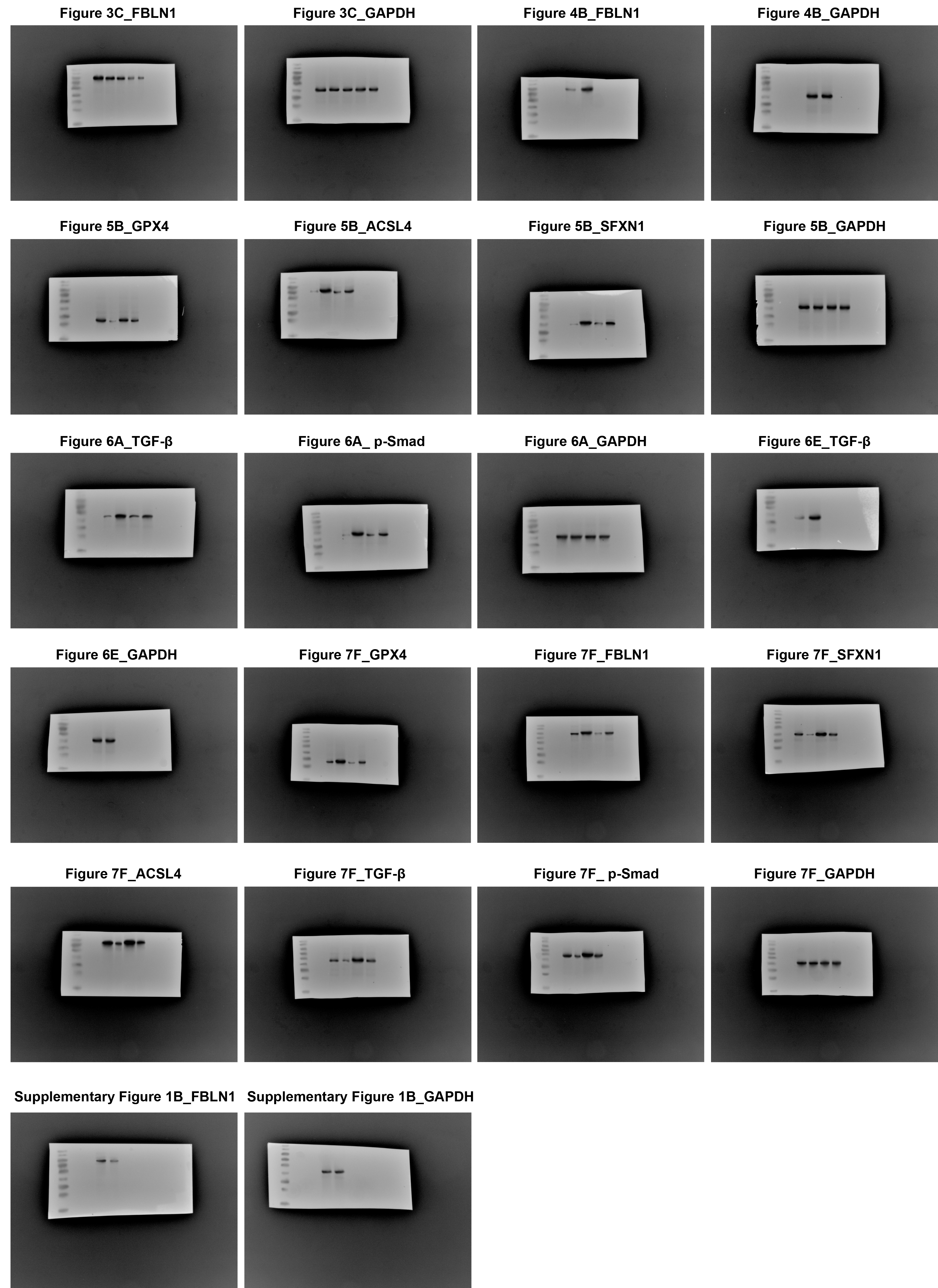

Supplement: S1 Raw images — (TIF) [file pone.0314750.s002.tif]
